# The prognostic marker NRIP1 is associated with tumor progression and immune infiltration in acute myeloid leukemia: NRIP1 is associated with tumor progression and immune infiltration

**DOI:** 10.3724/abbs.2025197

**Published:** 2025-11-04

**Authors:** Xunxun Zhu, Mingyan Zhang, Jingjing Zhang, Yanling Tao, Hao Zhang

**Affiliations:** 1 Medical Research Center Affiliated Hospital of Jining Medical University Jining 272000 China; 2 Postdoctoral Mobile Station of Shandong University Jinan 250000 China; 3 Key Laboratory of Cell and Biomedical Technology of Shandong Province Jining 272000 China; 4 Department of Hematology Affiliated Hospital of Jining Medical University Jining 272000 China; 5 Jining Key Laboratory of Hematopoietic Stem Cell Transplantation and Immunology Jining 272000 China; 6 Department of Pediatric Hematology Affiliated Hospital of Jining Medical University Jining 272000 China

**Keywords:** NRIP1, acute myeloid leukemia (AML), immune infiltration, prognostic biomarker

## Abstract

Acute myeloid leukemia (AML) is a clinically aggressive hematologic malignancy characterized by high relapse rates and treatment resistance, highlighting the need for novel biomarkers to improve clinical outcomes. In this study, we explore the roles of nuclear receptor-interacting protein 1 (NRIP1) in AML, focusing on its associations with tumor progression and immune infiltration. Analysis of public AML gene expression datasets reveals that NRIP1 expression is significantly increased in AML patients. Those with high NRIP1 expression have markedly shorter overall survival than those with low expression. Furthermore, NRIP1 expression is significantly associated with the infiltration of diverse immune cells, including B cells, dendritic cells, T cells, mast cells, eosinophils, and T helper cells, suggesting that NRIP1 may be a regulator of immune cell infiltration. Functional enrichment analysis indicates that NRIP1 and its interacting partners are involved in tumorigenesis, immune microenvironment remodeling, and metabolic reprogramming. Survival analysis confirms the prognostic value of NRIP1. Importantly, functional validation in AML cell lines confirms that *NRIP1* knockdown suppresses proliferation and induces apoptosis. Our study identifies NRIP1 as a multifaceted regulator that promotes AML by driving tumor progression, regulating immune cell infiltration, and modulating ferroptosis, highlighting its role as a novel prognostic biomarker.

## Introduction

Acute myeloid leukemia (AML) is a heterogeneous hematopoietic malignancy characterized by clonal expansion of hematopoietic stem cells, impaired differentiation, and evasion of apoptosis [1]. Despite advances in treatment modalities such as chemotherapy, hematopoietic stem cell transplantation, and immunotherapy, the 5-year survival rate for adult AML patients remains dismal at approximately 24% [2]. These limitations underscore the urgent need for a deeper understanding of AML pathogenesis and the identification of novel prognostic biomarkers and therapeutic targets.

The initiation, progression, and relapse of AML are driven not only by acquired genetic mutations [3] but also by the dynamic interplay between leukemic cells and the tumor microenvironment (TME) [ 4, 5] . The bone marrow TME in AML, comprising an altered extracellular matrix, immunosuppressive immune cells, and soluble factors, fosters a protective niche that undermines conventional and immune-based therapies. Consequently, overcoming this immunosuppressive environment is crucial for enhancing therapeutic efficacy.

Nuclear receptor-interacting protein 1 (NRIP1), also known as RIP140, is a multifaceted transcriptional coregulator that modulates the activity of multiple transcription factors and nuclear receptors [6]. Its roles as either an oncogene or a tumor suppressor have been documented in several solid tumors, including breast [ 7, 8] , colon [9], and liver [10] cancers. For instance, in breast cancer, NRIP1 promotes tumor progression by regulating interferon gamma signaling [7] and glycolytic metabolism [8]. Previous studies have demonstrated that NRIP1 is highly expressed in hematopoietic stem cells and that its expression level is correlated with prognosis in patients with chronic lymphocytic leukemia (CLL) [11]. Furthermore, the oncogenic potential of NRIP1 can be unleashed not only through transcriptional upregulation but also through genetic alterations, as exemplified by the recent identification of the NRIP1::PDGFRB fusion gene in pediatric Philadelphia chromosome-like acute lymphoblastic leukemia (Ph-like ALL) [12]. This fusion constitutively activates the tyrosine kinase PDGFRB, driving leukemogenesis and thereby representing a novel therapeutic target. Moreover, a NRIP1-MIR99AHG fusion transcript accompanied by a genomic inversion was recently discovered in patients with chronic myelomonocytic leukemia (CMML), suggesting its potential role in the biology and progression of this disease [13]. These findings highlight that NRIP1 is not merely a passive biomarker but also a central, active driver of leukemogenesis through structural rearrangements.

Furthermore, therapy resistance remains a major hurdle in AML treatment. Recent studies have highlighted the critical roles of diverse signaling pathways and metabolic reprogramming in mediating chemoresistance. For instance, the AHR signaling pathway has been shown to contribute to cytarabine resistance by enhancing mitochondrial oxidative phosphorylation [14]. These findings highlight the complexity of resistance mechanisms in AML and emphasize the need to identify novel biomarkers and targets to overcome this challenge.

However, despite these compelling insights, the function, clinical significance, and mechanistic roles of NRIP1 in AML remain largely unexplored. Given its involvement in critical cellular processes across other cancers, we hypothesized that NRIP1 might also play a pivotal role in AML pathogenesis, influencing clinical outcomes, immune evasion, and therapy response. To address this hypothesis, we performed integrated bioinformatics analyses of public datasets (TCGA and GEO) and *in vitro* functional experiments. This study aimed to comprehensively investigate the expression landscape, prognostic value, and potential roles of NRIP1 in immune infiltration and ferroptosis regulation in AML, thereby elucidating its potential as a novel biomarker.

## Materials and Methods

### Data processing

Primary RNA-seq data (TPM values) and corresponding clinical annotations for 173 AML patients were obtained from The Cancer Genome Atlas (TCGA-LAML) database. Table 1 presents the baseline characteristics of the AML patients included in the analysis, excluding 23 samples with incomplete clinical annotations. The expression data for the control group were obtained from the Genotype-Tissue Expression (GTEx) project. To validate our findings, we acquired microarray expression profiles (GSE37642-GPL96 platform) and clinical outcomes for an additional 422 AML patients from the Gene Expression Omnibus (GEO) database. We performed bioinformatics analyses using the Xiantao Academic platform ( https://www.xiantaozi.com), a comprehensive bioinformatics toolkit for cancer genomics research.

**Table:** **Table 1** Relationship between NRIP1 expression and clinicopathological features in the TCGA database

Characteristics	Low expression of NRIP1	High expression of NRIP1	*P* value
*n*	75	75	
Gender, *n* (%)			0.071
Female	39 (26%)	28 (18.7%)	
Male	36 (24%)	47 (31.3%)	
Missing	0	0	
Race, *n* (%)			1.000
Asian	1 (0.7%)	0 (0%)	
White	68 (45.6%)	67 (45%)	
Black or African American	6 (4%)	7 (4.7%)	
Missing	0	1	
Age, n (%)			0.408
≤ 60	46 (30.7%)	41 (27.3%)	
> 60	29 (19.3%)	34 (22.7%)	
Missing	0	0	
WBC count (×10 ^9^/L), *n* (%)			0.368
≤ 20	35 (23.5%)	41 (27.5%)	
> 20	39 (26.2%)	34 (22.8%)	
Missing	1	0	
BM blasts (%), *n* (%)			0.867
≤ 20	30 (20%)	29 (19.3%)	
> 20	45 (30%)	46 (30.7%)	
Missing	0	0	
PB blasts (%), *n* (%)			0.034
≤ 70	29 (19.3%)	42 (28%)	
> 70	46 (30.7%)	33 (22%)	
Missing	0	0	
Cytogenetic risk, *n* (%)			< 0.001
Favorable	22 (14.9%)	8 (5.4%)	
Intermediate	44 (29.7%)	38 (25.7%)	
Poor	8 (5.4%)	28 (18.9%)	
Missing	1	1	
FAB classifications, *n* (%)			< 0.001
M0	2 (1.3%)	13 (8.7%)	
M1	17 (11.4%)	18 (12.1%)	
M2	14 (9.4%)	24 (16.1%)	
M3	14 (9.4%)	0 (0%)	
M4	18 (12.1%)	11 (7.4%)	
M5	10 (6.7%)	5 (3.4%)	
M6	0 (0%)	2 (1.3%)	
M7	0 (0%)	1 (0.7%)	
Missing	0	1	
FLT3 mutation, *n* (%)			0.133
Negative	47 (32.2%)	54 (37%)	
Positive	27 (18.5%)	18 (12.3%)	
Missing	1	3	
IDH1 R132 mutation, *n* (%)			0.811
Negative	67 (45.3%)	68 (45.9%)	
Positive	6 (4.1%)	7 (4.7%)	
Missing	2	0	
IDH1 R140 mutation, *n* (%)			0.079
Negative	66 (44.6%)	70 (47.3%)	
Positive	9 (6.1%)	3 (2%)	
Missing	0	2	
IDH1 R172 mutation, *n* (%)			1.000
Negative	74 (50%)	72 (48.6%)	
Positive	1 (0.7%)	1 (0.7%)	
Missing	0	2	
RAS mutation, *n* (%)			0.731
Negative	70 (47%)	71 (47.7%)	
Positive	5 (3.4%)	3 (2%)	
Missing	0	1	
NPM1 mutation, *n* (%)			0.033
Negative	53 (35.6%)	63 (42.3%)	
Positive	22 (14.8%)	11 (7.4%)	
Missing	0	1	

### Survival analysis

Survival analysis was performed on the TCGA-LAML and GSE37642-GPL96 datasets via the Kaplan-Meier method. The R package “survival” (version 3.3.1) was used to perform proportional hazards assumption testing and fit survival regression, with the results visualized using the “survminer (version 0.4.9)” package and the “ggplot2 (version 3.4.4)” package. To assess the prognostic significance of NRIP1, patients were stratified into high- and low-expression groups on the basis of median NRIP1 mRNA levels using a standardized threshold.

### Patient specimens

To establish clinically relevant findings, we prospectively collected bone marrow samples from 35 newly diagnosed AML patients and 20 healthy controls from the Affiliated Hospital of Jining Medical University (Jining, China). All participants met stringent inclusion criteria and provided written informed consent as stipulated in the Declaration of Helsinki under ethics approval (No. 2021-11-C020). Mononuclear cells were isolated by density gradient centrifugation using Ficoll density gradient centrifugation (Solarbio, Beijing, China) and stored at –80°C.

### Differentially expressed gene analysis

Samples from the TCGA-LAML cohort were stratified into high- and low-NRIP1 expression groups on the basis of median expression values. We identified differentially expressed genes (DEGs) via the “DESeq2” package (version 1.36.0) in R. Genes with an adjusted *P* value < 0.05 and a |log2fold change| > 1 were considered statistically significant. We subsequently examined the correlation between NRIP1 expression levels and the top five most significantly up- and down-regulated DEGs using Spearman’s rank correlation analysis. The global differential expression profile was visualized via volcano plots, with thresholds indicating statistical significance (adjusted *P* value) and the magnitude of change (fold change).

### Functional enrichment analysis

We carried out functional enrichment analysis on the DEGs using the “clusterProfiler” package (version 4.4.4) [15] in R, which incorporates Gene Ontology (GO) biological processes, Kyoto Encyclopedia of Genes and Genomes (KEGG) pathways, and gene set enrichment analysis (GSEA). For GSEA, we utilized the Molecular Signatures Database (MSigDB) collection c2.cp.all.v2022.1.Hs.symbols.gmt (All Canonical Pathways, 3050). Significant enrichments were defined as those meeting both an adjusted *P* value < 0.05 and a false discovery rate (FDR) < 0.25. The results were visualized via ggplot2 (version 3.4.0), with dot plots displaying enrichment scores and gene ratio metrics for GO/KEGG analyses and ridge plots illustrating normalized enrichment scores for GSEA pathways.

### Protein-protein interaction network analysis

We constructed protein-protein interaction (PPI) networks with the STRING database (version 11.5). A minimum interaction confidence score threshold of 0.7 was applied to select high-confidence interactions, which identified 15 functionally associated partners of NRIP1 [16]. Complementary analysis was performed using GeneMANIA ( http://genemania.org), which integrates multiple data types, including co-expression, physical interaction, and genetic interaction data, to predict gene function and construct comprehensive interaction networks [17].

### Gene mutations

To characterize the genomic alterations of NRIP1 in AML, we interrogated mutation profiles through cBioPortal ( https://www.cbioportal.org/) using two independent AML datasets [18]. The analysis integrated three core elements: genomic alteration profiling of NRIP1 (amplifications and deletions), subtype-specific frequency analysis across molecular subgroups, and clinical outcome correlation with genomic status. All analyses employed the built-in statistical framework of cBioPortal, with significance thresholds set at *P*  < 0.05.

### Immune infiltration analysis

Immune cell infiltration profiles were quantified using single-sample gene set enrichment analysis (ssGSEA) implemented in the R package GSVA (version 1.46.0), which calculates enrichment scores for 24 functionally defined immune cell subsets [19]. Spearman’s rank correlation analysis was used to evaluate associations between NRIP1 expression levels and immune infiltration patterns [20]. Differences in immune cell infiltration between groups with high and low NRIP1 expression were analyzed using the Wilcoxon rank-sum test.

### Cell culture and transfection

The human leukemia cell lines Molm-13 and Thp-1 were obtained from Procell Biotechnology (Wuhan, China), while HL-60, MV-4-11, OCI-AML3, and HEK293T cells were acquired from the Medical Research Center at Jining Medical University Affiliated Hospital. Molm-13, Thp-1, HL-60, and MV-4-11 cells were routinely cultured in RPMI-1640 (Gibco, Carlsbad, USA) supplemented with 10% FBS (Gibco). OCI-AML3 cells were cultured in RPMI-1640 supplemented with 20% FBS, while HEK293T cells were grown in DMEM (Gibco) supplemented with 10% FBS. NRIP1-specific siRNA duplexes and a scrambled negative control siRNA were designed and synthesized by GenePharma (Shanghai, China). The sequences of siRNAs used were as follows: si *NRIP1-1*, sense (5′-GGUUGACAGUGUGCCUAAATT-3′) and antisense (5′-UUUAGGCACACUGUCAACCTT-3′); si *NRIP1-2*, sense (5′-GAUGUGCACCAGGAUUCUATT-3′) and antisense (5′-UAGAAUCCUGGUGCACAUCTT-3′); si *-*NC, sense (5′-UUCUCCGAACGUGUCACGUTT-3′) and antisense (5′-ACGUGACACGUUCGGAGAATT-3′). For transfection, cells were seeded in 6-well plates and transfected using Lipofectamine RNAiMAX (Invitrogen, Carlsbad, USA) according to the manufacturer’s protocol.

### Quantitative reverse-transcription PCR

Total RNA was isolated from cultured cells using TRIzol reagent (Invitrogen) following the manufacturer’s protocol. For cDNA synthesis, 1 μg of total RNA was reverse transcribed in a 20-μL reaction volume using HiScript III RT SuperMix (Vazyme, Nanjing, China). Quantitative real-time PCR was performed on the QuantStudio 6 Flex system (Applied Biosystems, Foster City, USA) using ChamQ Universal SYBR qPCR Master Mix (Vazyme). Gene expression levels were quantified using the 2 ^‒ΔΔCt^ method, with *β-actin* serving as the endogenous reference gene for normalization. All reactions were performed in technical triplicates, with melt curve analysis confirming amplification specificity. The sequences of primers used were as follows: *NRIP1*, forward primer (5′-CCAGCCCAAAATGAAGGTGC-3′) and reverse primer (5′-GTTTGCTGGGTCTCTGCTCT-3′); *β-actin*, forward primer (5′-CTCGCCTTTGCCGATCC-3′) and reverse primer (5′-ATCCTTCTGACCCATGCCC-3′).

### Western blot analysis

The cells were lysed using RIPA buffer (Beyotime, Shanghai, China) supplemented with protease inhibitors (Roche, Basel, Switzerland) for 30 min on ice. Protein concentrations were determined using a BCA assay kit (Beyotime) prior to separation on 10% SDS-polyacrylamide gels. Electrophoresed proteins were transferred to nitrocellulose membranes (Millipore, Billerica, USA) using a semi-dry transfer system (Bio-Rad, Hercules, USA). The membranes were blocked with protein-free rapid blocking buffer (Yamei, Hefei, China) for 15 min at room temperature and then incubated overnight at 4°C with primary antibodies against NRIP1 (#ab42126, 1:1000; Abcam, Cambridge, UK), GPX4 (#59735, 1:1000; Cell Signaling Technology, Danvers, USA), SLC7A11 (#ab307601, 1:1000; Abcam), ALOX15 (#ab244205, 1:1000; Abcam), and β-actin (#ab241153, 1:500; Abcam). After washing, the membranes were incubated with HRP-conjugated secondary antibodies (#ab97051, 1:20,000; Abcam) for 1 h at room temperature. The protein bands were visualized using enhanced chemiluminescence substrate (Beyotime) and quantified using ImageJ software.

### Cell proliferation

Cell proliferation was assessed by CCK-8 assay. Briefly, cells were seeded in 96-well plates at a density of 5 × 10³ cells/well and cultured under standard conditions (37°C, 5% CO _2_). At the designated time points (0, 24, 48, 72 and 96 h), 10 μL of CCK-8 reagent (Glpbio, Montclair, USA) was added to each well, followed by incubation for 2 h. We measured the absorbance at 450 nm (with a reference wavelength of 650 nm) on a Synergy H1 microplate reader (BioTek, Winooski. USA).

### Apoptosis analysis

Apoptosis was quantified 48 h post-transfection using an Annexin V-FITC/PI dual-staining kit (Beyotime). The cells were washed twice with ice-cold PBS and resuspended in 300 μL of 1× binding buffer. Following incubation with 5 μL of Annexin V-FITC and 10 μL of propidium iodide (PI) for 15 min at room temperature in the dark, the samples were immediately analyzed on a CytoFLEX flow cytometer (Beckman Coulter, Pasadena, USA). The Annexin V ^+^/PI ^–^ population represents early apoptotic cells, whereas the Annexin V ^+^/PI ^+^ population represents late apoptotic cells. The data were analyzed using FlowJo software with standardized gating strategies.

### Statistical analysis

All the statistical analyses were conducted with IBM SPSS Statistics 26 and R software (version 4.2.1). Continuous variables were compared via Wilcoxon rank-sum tests for unpaired samples and paired *t* tests for matched samples, with normality assessed by Shapiro-Wilk tests. Associations between NRIP1 expression and clinical parameters were evaluated through the Wilcoxon test for categorical variables and Spearman’s rank correlation for continuous variables. All tests were two-tailed, with *P*  < 0.05 considered statistically significant. Data are presented as the mean ± SEM. Data visualization was generated using ggplot2 (version 3.4.0) in R.

## Results

### NRIP1 is highly expressed in AML patients and associated with adverse clinical features

**Figure 1:**
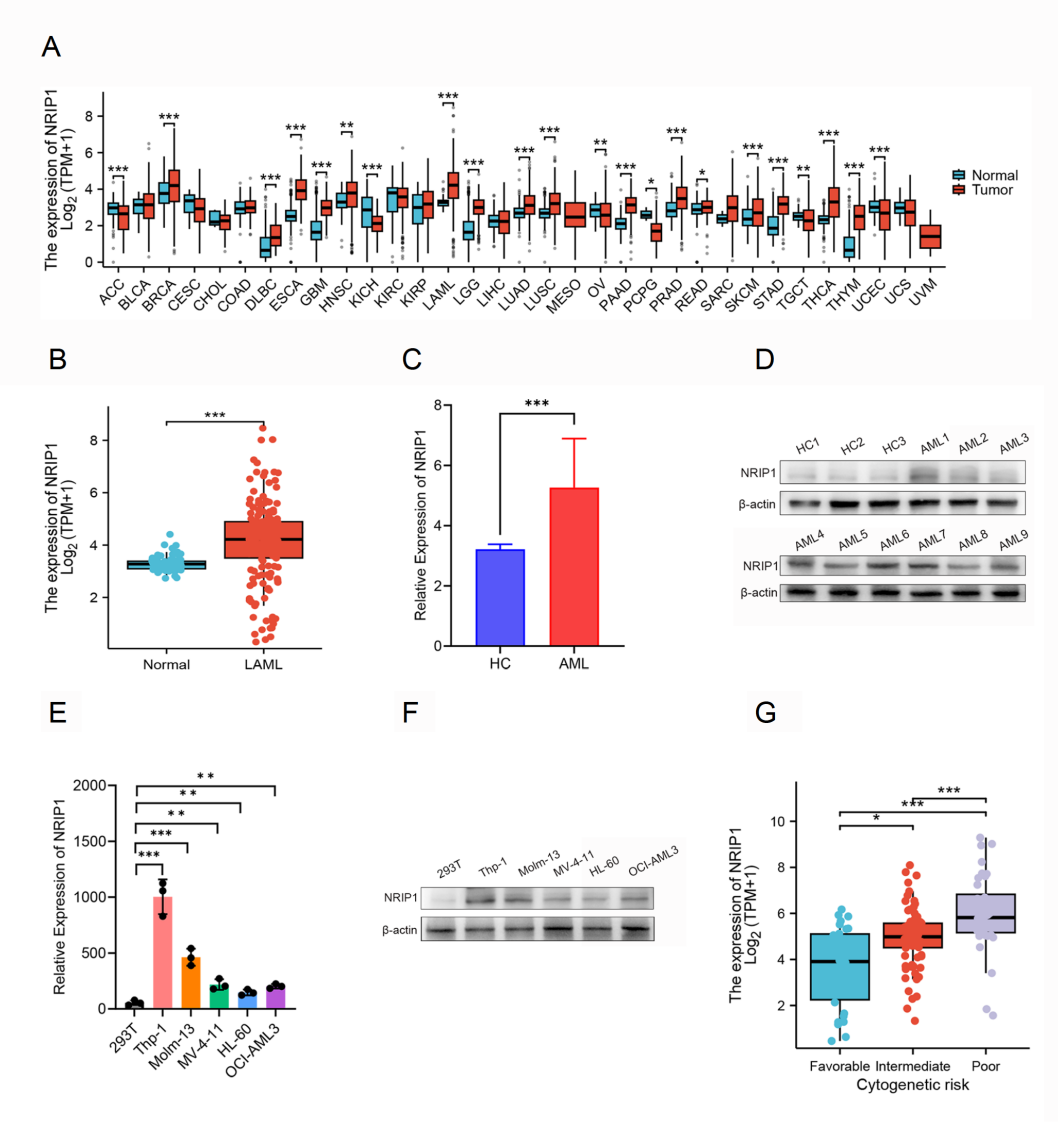
Figure 1 NRIP1 expression across malignancies and its clinicopathological relevance in AML (A) Comparison of NRIP1 mRNA expression in tumor samples (red) and matched normal tissues (blue) across multiple cancer types. (B) NRIP1 mRNA levels in TCGA AML samples (n = 173) versus GTEx normal controls (n = 70). (C) qRT-PCR analysis of NRIP1 mRNA expression in AML patients (n = 35) versus healthy donors (HCs, n = 20). (D) Western blot analysis of NRIP1 protein expression in AML patients (n = 9) and HCs (n = 3). (E,F) qRT-PCR (E) and western blot analysis (F) of NRIP1 expression in a panel of human leukemia cell lines. 293T cells were used as a control. (G) NRIP1 expression stratified by cytogenetic risk subgroup in the TCGA-LAML cohort. TCGA, The Cancer Genome Atlas; GTEx, Genotype Tissue Expression Project. *P < 0.05, **P < 0.01, ***P < 0.001.

Analysis of the TCGA and GTEx datasets consistently revealed that NRIP1 was upregulated in multiple malignancies, including invasive breast carcinoma, esophageal carcinoma, thyroid carcinoma, and thymoma, compared with matched normal tissues ( Figure 1A). Consistently, NRIP1 expression was significantly elevated in AML samples compared with normal controls ( Figure 1B). This finding was confirmed in our independent clinical cohort, where the mRNA levels of *NRIP1* were markedly increased in AML patients compared with healthy controls ( Figure 1C). To confirm these findings at the protein level, we performed western blot analysis of NRIP1 in a subset of samples (9 from AML patients and 3 from healthy controls), which revealed a corresponding increase in NRIP1 protein expression in AML samples ( Figure 1D). Consistent with these observations, qRT-PCR and western blot analysis confirmed NRIP1 overexpression in AML cell lines, with the highest levels observed in Thp-1 and Molm-13 cells ( Figure 1E,F). Accordingly, we selected these two cell lines for subsequent functional studies.

We found that high NRIP1 expression was associated with adverse disease features, especially poorer cytogenetic risk profiles ( Figure 1G and Table 1). We further investigated NRIP1 expression across different subtypes of AML via the French-American-British (FAB) classification. As shown in Supplementary Figure S1, NRIP1 expression varied among subtypes, with notably higher expression observed in M0 subtype. Given that the M0 subtype is often associated with a primitive, undifferentiated phenotype, this expression pattern suggests a potential role in maintaining the primitive state and pathogenicity of leukemia stem cells by regulating cell metabolism and differentiation. Univariate logistic regression identified NRIP1 as significantly associated with adverse clinical characteristics, particularly for cytogenetic risk (OR = 5.022, 95% CI: 2.101–12.001, *P*  < 0.001), NPM1 mutation status (OR = 0.421, 95% CI: 0.187–0.946, *P* = 0.036), and peripheral blast percentage (OR = 0.495, 95% CI: 0.258–0.950, *P* = 0.034) ( Table 2). Collectively, these results identify NRIP1 as a clinically relevant biomarker in AML.

**Table:** **Table 2** Logistic regression analysis of NRIP1 expression

Characteristics	Total ( *n*)	OR (95% CI)	*P* value
Gender (male vs female)	150	1.818 (0.948–3.488)	0.072
Age (> 60 vs ≤ 60)	150	1.315 (0.687–2.520)	0.409
WBC count (x10 ^9^/L) (> 20 vs ≤ 20)	149	0.744 (0.391–1.417)	0.369
PB blasts (%) (> 70 vs ≤ 70)	150	0.495 (0.258–0.950)	0.034
FLT3 mutation (positive vs negative)	146	0.580 (0.284–1.184)	0.135
RAS mutation (positive vs negative)	149	0.592 (0.136–2.570)	0.484
NPM1 mutation (positive vs negative)	149	0.421 (0.187–0.946)	0.036
Cytogenetic risk (poor vs favorable&intermediate)	148	5.022 (2.101–12.001)	< 0.001

### Prognostic significance of NRIP1 in AML

**Figure 2:**
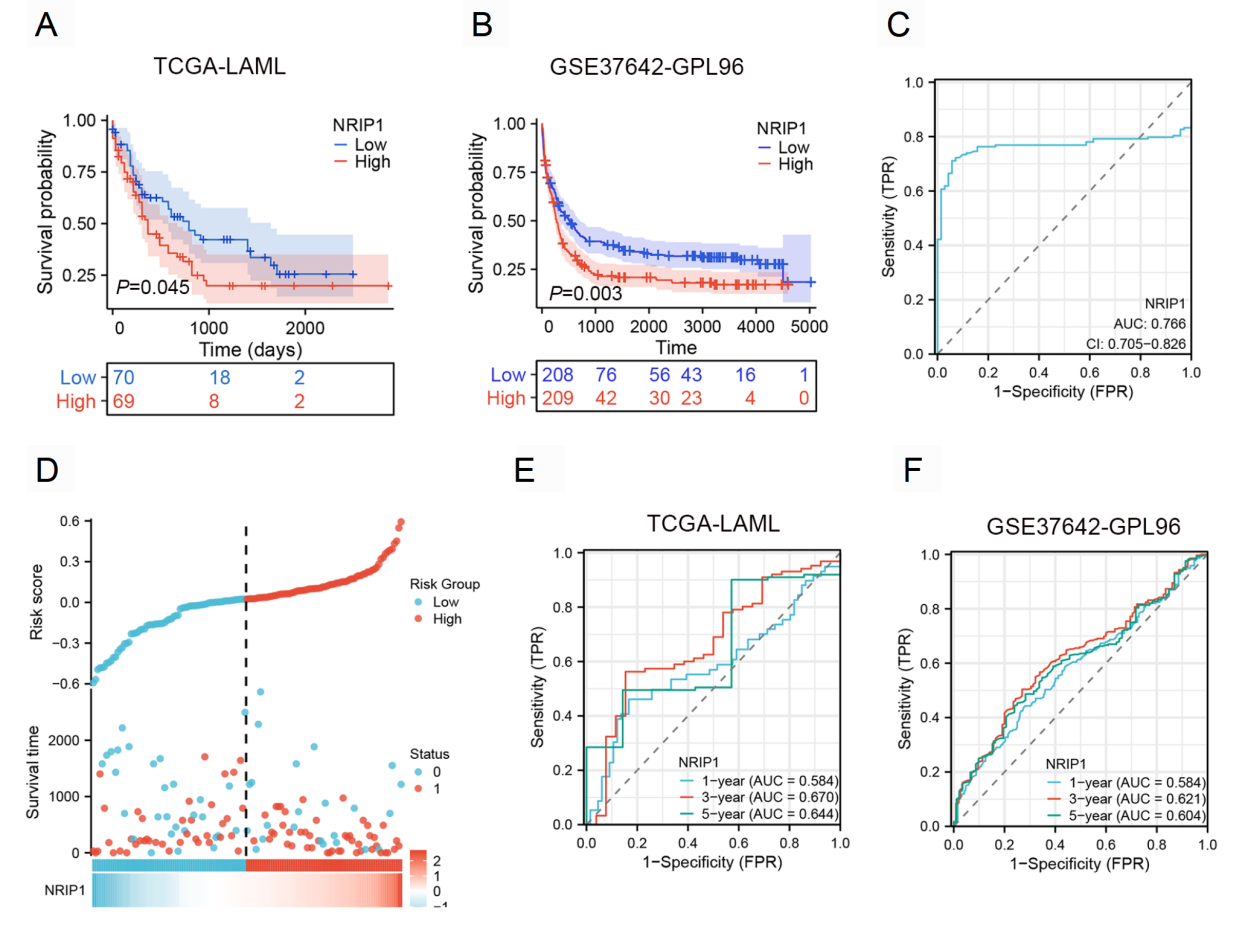
Figure 2 NRIP1 expression predicts poor prognosis in AML patients (A) Kaplan-Meier curves comparing the OS of patients in the TCGA-LAML cohort stratified by high (red line) and low (blue line) NRIP1 expression. (B) Validation of the prognostic value of NRIP1 in an independent cohort from the GEO dataset (GSE37642-GPL96). (C) Diagnostic ROC curves in TCGA-LAML. (D) Risk score, survival time distribution, and gene expression heatmap of NRIP1 in the TCGA cohort. (E,F) Time-dependent ROC analysis for the prediction of 1-, 3-, and 5-year OS in the TCGA cohort (E) and the GSE37642-GPL96 cohort (F).

Given that NRIP1 is overexpressed in AML, we evaluated its prognostic value. Patients were classified into high- and low-NRIP1 expression groups via the “surv-cutpoint” algorithm for optimal stratification [21]. Kaplan-Meier analysis revealed that patients with high NRIP1 expression had significantly shorter overall survival (OS) than those with low NRIP1 expression in both the TCGA-LAML cohort ( *P* = 0.045) and the independent GEO validation cohort (GSE37642-GPL96, *P* = 0.003; Figure 2A,B). Moreover, receiver operating characteristic (ROC) analysis confirmed the favorable diagnostic performance of NRIP1 expression (AUC = 0.766; Figure 2C). As detailed in Supplementary Figure S2, the consistent and significant AUC values across different cytogenetic risk factors and mutation statuses (FLT3, NPM1, *etc*.) confirmed the robust predictive capacity of NRIP1. Furthermore, a risk score model incorporating NRIP1 expression effectively stratified patients into distinct prognostic groups, with high-risk patients experiencing significantly shorter event-free survival ( Figure 2D). The prognostic utility of NRIP1 was additionally validated by time-dependent ROC curve analysis, which revealed consistent predictive power for 1-, 3-, and 5-year survival rates ( Figure 2E,F). These findings underscore the role of NRIP1 in AML prognosis.

### Differential gene expression analysis reveals NRIP1-associated molecular pathways

**Figure 3:**
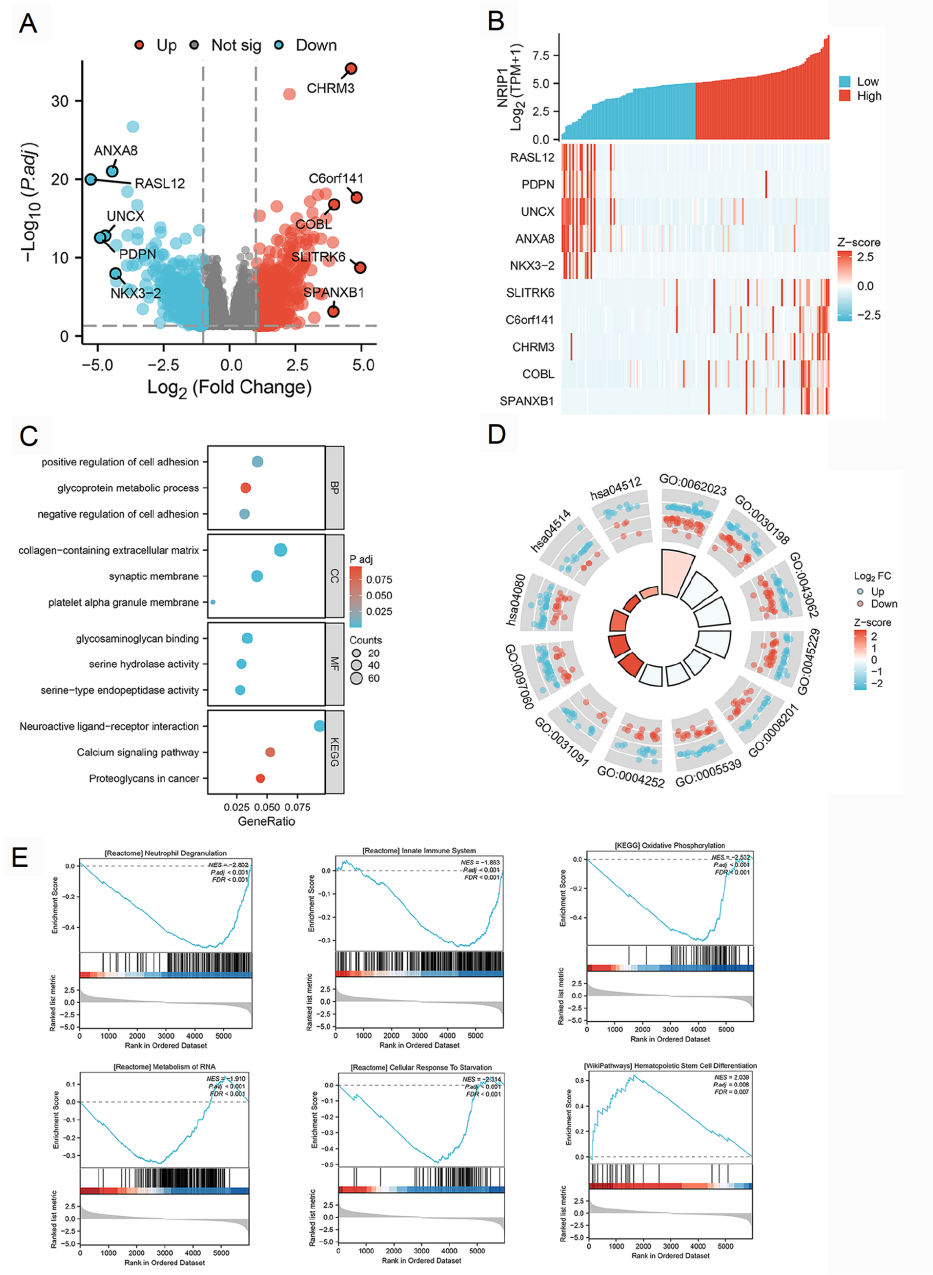
Figure 3 Differential gene expression and functional enrichment associated with NRIP1 in AML (A) Volcano plot of DEGs between the NRIP1-high and NRIP1-low AML groups (|log2fold change| > 1, adjusted P < 0.05). (B) Heatmap of the top five upregulated and downregulated DEGs correlated with NRIP1 expression. (C) Bubble plot of GO and KEGG enrichment for DEGs. (D) Circular visualization of enriched GO/KEGG terms. (E) GSEA highlighting pathways associated with high NRIP1 expression. GO: Gene Ontology; KEGG: Kyoto Encyclopedia of Genes and Genomes; GSEA: Gene Set Enrichment Analysis.

To investigate the biological functions of NRIP1 in AML, we conducted differential gene expression analysis. This comprehensive analysis of the high- and low-NRIP1-expressing AML cohorts revealed 1140 DEGs (adjusted *P*  < 0.05, |log2 fold change| > 1), including 742 upregulated and 398 downregulated genes ( Figure 3A). Among these genes, the most differentially expressed genes (RASL12, PDPN, UNCX, ANXA8, NKX3-2, SLITRK6, C6orf141, CHRM3, COBL, and SPANXB1) were significantly correlated with NRIP1 expression levels according to hierarchical clustering analysis ( Figure 3B). Functional annotation indicated that NRIP1-associated genes were mainly enriched in biological processes involved in the regulation of cell adhesion and glycoprotein metabolism. These genes were also localized to extracellular matrix components and synaptic membranes and were involved in glycosaminoglycan binding and serine-type enzymatic activities ( Figure 3C,D).

Additionally, GSEA of canonical pathways revealed that low NRIP1 expression was enriched for pathways such as neutrophil degranulation, innate immunity, oxidative phosphorylation, RNA metabolism and the cellular response to starvation, whereas high NRIP1 expression was enriched for gene sets related to hematopoietic differentiation programs ( Figure 3E).

These findings suggest that NRIP1 may regulate AML pathogenesis through the modulation of cell-matrix interactions, immune surveillance, and metabolic reprogramming, indicating its pleiotropic functions in disease progression. The coordinated dysregulation of these fundamental biological processes underscores the multifaceted involvement of NRIP1 in AML pathophysiology.

### PPI network identifies key NRIP1-associated proteins

**Figure 4:**
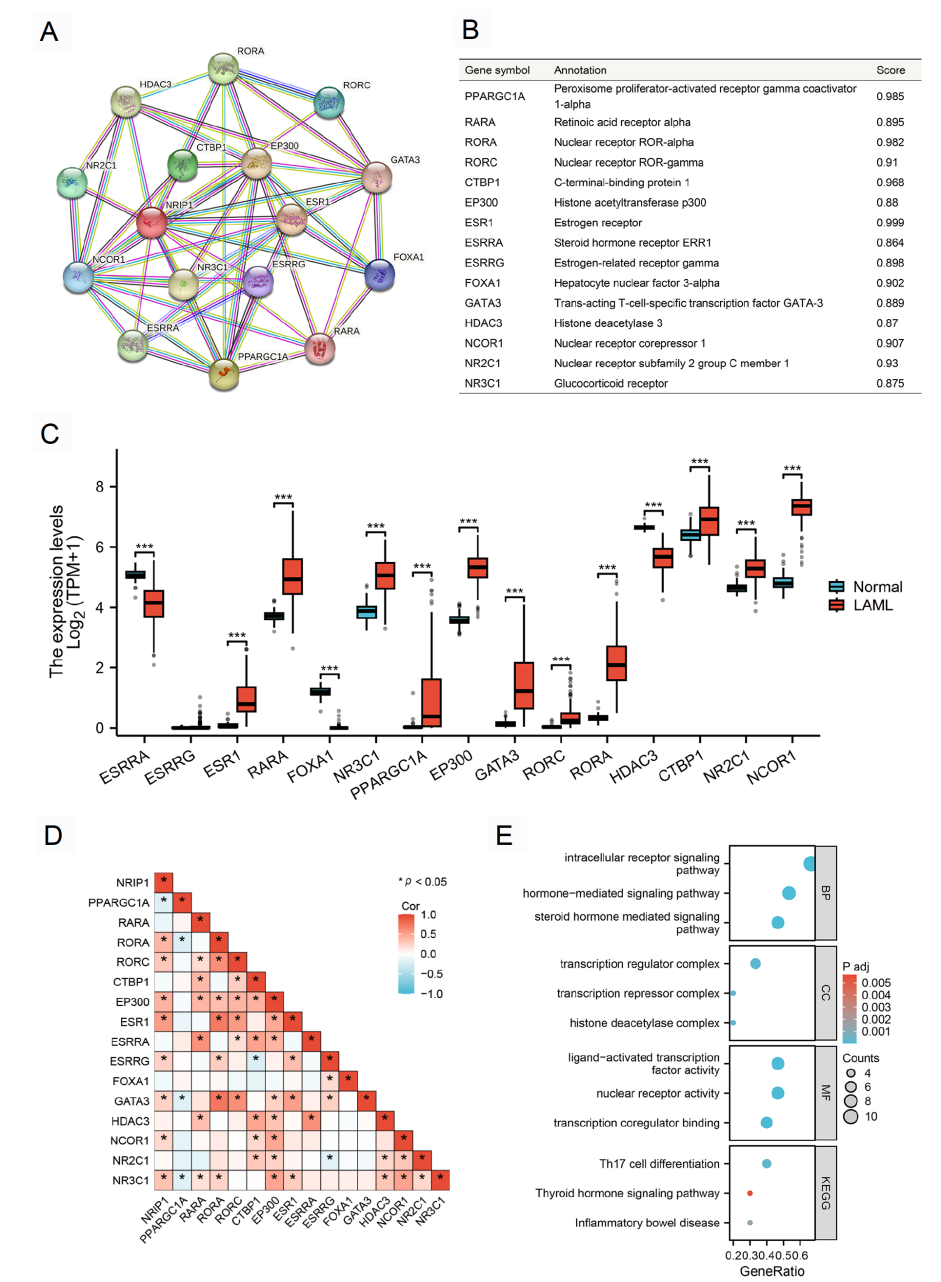
Figure 4 PPI network and functional annotation of NRIP1-associated genes (A) PPI network of NRIP1-related genes (confidence score > 0.7). (B) Correlation matrix and annotation of 15 NRIP1-related genes. (C) mRNA expression levels of NRIP1-related genes in TCGA-LAML samples (n = 173) compared with normal samples from the GTEx database (n = 70). (D) Correlation between NRIP1 and related genes. (E) GO/KEGG enrichment of NRIP1-related genes. ***P < 0.001.

**Figure 5:**
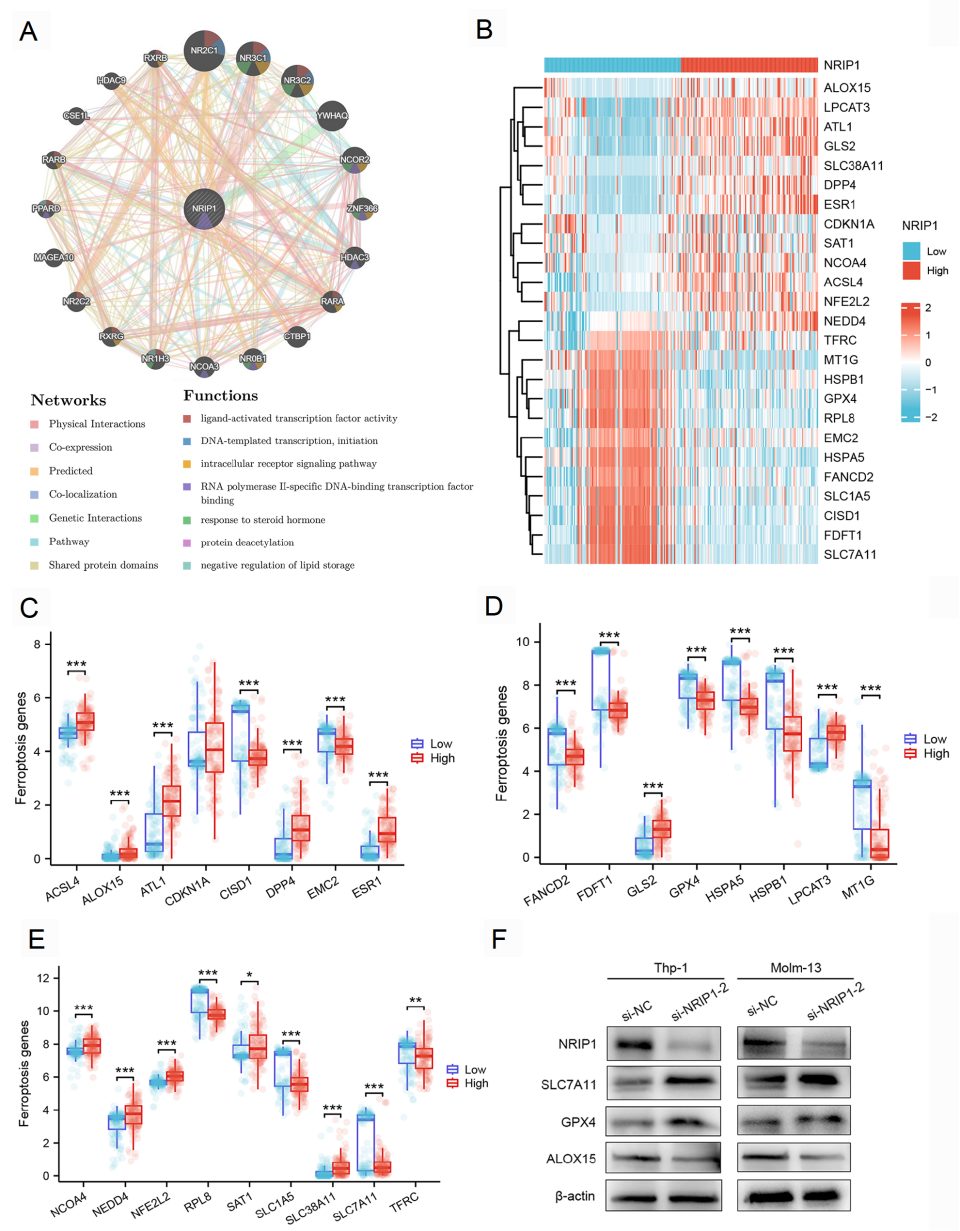
Figure 5 NRIP1 is associated with the regulation of ferroptosis in AML (A) GeneMANIA network illustrating the functional interactions of NRIP1. (B) Heatmap of correlations between NRIP1 and 25 ferroptosis-related genes in the TCGA-LAML dataset (n = 173). (C–E) Boxplots comparing the expressions of ferroptosis drivers (ACSL4, ALOX15) and suppressors (GPX4, SLC7A11) in the NRIP1-high vs NRIP1-low groups. (F) Western blot analysis of GPX4, SLC7A11, and ALOX15 protein levels in AML cells transfected with si-NC or si-NRIP1-2. *P < 0.05, **P < 0.01, ***P < 0.001.

Next, we constructed a PPI network based on the DEGs using the STRING database to identify NRIP1-interacting partners. Through systematic analysis of the STRING protein interaction database (confidence score > 0.7), we identified 15 high-confidence NRIP1-interacting partners, including nuclear receptors (RARA, RORA, RORC, and ESR1), transcriptional coregulators (PPARGC1A, CTBP1, EP300, and NCOR1), and epigenetic modifiers (HDAC3) ( Figure 4A,B,D). Comparative expression profiling revealed distinct patterns between AML and normal samples, with ESRRA, FOXA1, and HDAC3 showing higher expression in normal hematopoietic cells, while the majority of interactors (12/15) were significantly upregulated in AML ( Figure 4C). Through integrated GO/KEGG analysis, functional annotation revealed that these NRIP1-associated proteins predominantly participate in the intracellular receptor signaling pathway, nuclear receptor signaling pathways, and hormone-mediated signaling pathways ( Figure 4E). Additionally, GeneMANIA network analysis [22] corroborated these findings ( Figure 5A).

The network was particularly enriched for genes involved in ligand-activated transcription factor activity and transcription coregulator binding. Notably, some of these interacting proteins have established roles in hematopoietic malignancies. RARA [23] is a clinically validated therapeutic target in AML, whereas HDAC3 [24] and EP300 [25] have been identified as potential therapeutic targets in AML.

### NRIP1 is correlated with ferroptosis-related genes in AML

Ferroptosis is an iron-dependent form of regulated cell death distinct from apoptosis and necrosis [26]. Unlike normal cells, AML cells frequently exhibit increased iron uptake ( *e*. *g*., via upregulation of the transferrin receptor CD71) [27] and reduced iron storage ( *e*. *g*., decreased ferritin expression) [28], resulting in dysregulation of iron metabolism [29]. Studies have confirmed that certain genes are associated with the ferroptosis process in AML [ 30– 32] . Through systematic analysis of the TCGA-LAML cohort, we identified significant correlations between NRIP1 expression and the expression of 25 ferroptosis-related genes ( Figure 5B). A comprehensive correlation analysis confirmed these strong statistical relationships ( Supplementary Figure S3). Notably, NRIP1 was significantly positively correlated with pro-ferroptotic drivers, including ACSL4, ALOX15, and NCOA4, but was inversely correlated with ferroptosis suppressors, such as GPX4 and SLC7A11 ( Figure 5C–E). To experimentally validate the association between NRIP1 and ferroptosis-related gene expression, we examined the protein levels of key ferroptosis regulators after *NRIP1* knockdown in Thp-1 and Molm-13 cells. Western blot analysis confirmed that *NRIP1* silencing reproduced the expression patterns observed in the low-NRIP1 patient cohort ( Figure 5F). These results indicate that NRIP1 modulates the expression of critical ferroptosis regulators, strongly supporting its role in influencing ferroptosis in AML. Our finding is particularly intriguing in the context of recent evidence that iron overload drives chemoresistance in AML by inhibiting the TP53 pathway [33], collectively suggesting a complex interplay between iron metabolism, canonical apoptosis, and ferroptosis.

### Genomic alterations predict clinical outcomes in AML

**Figure 6:**
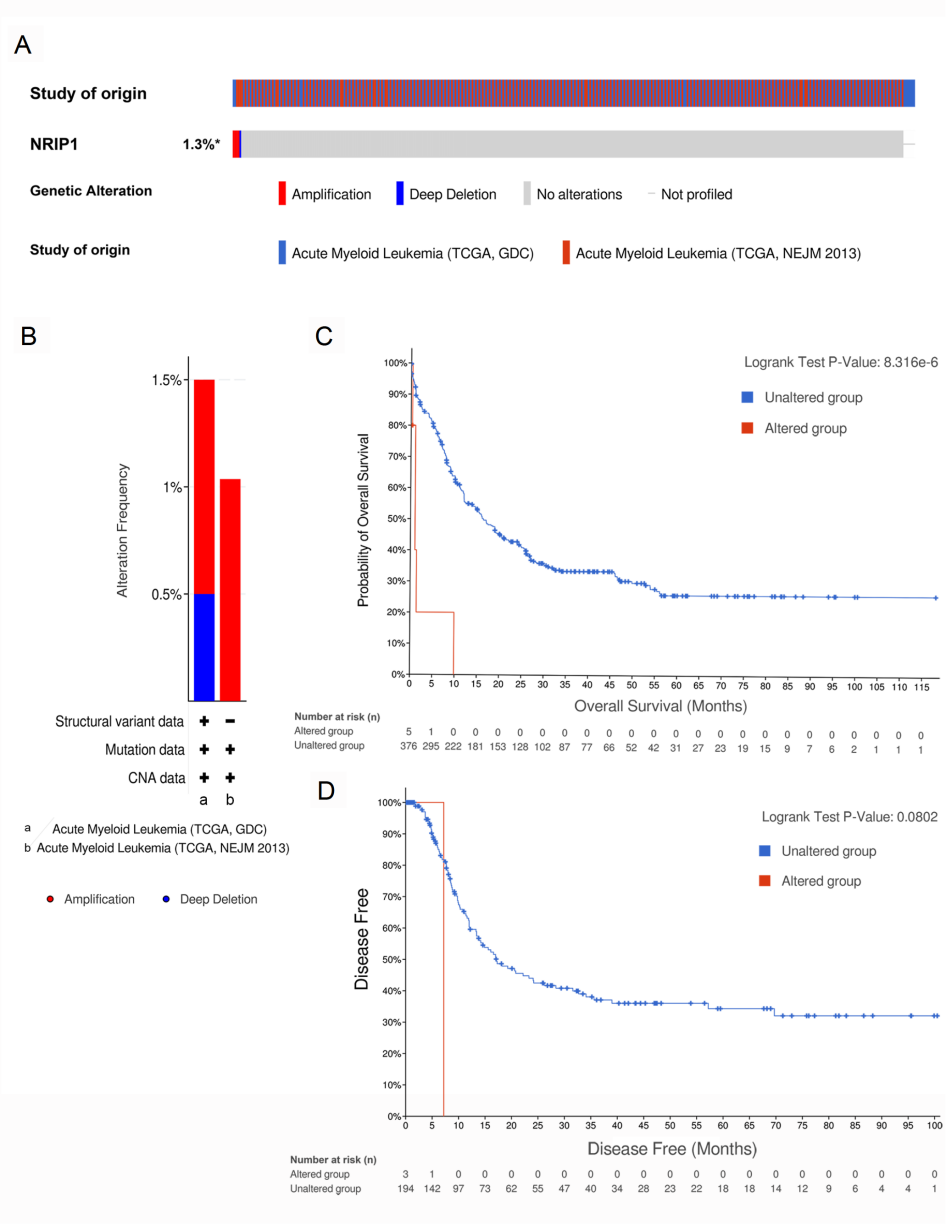
Figure 6 Genomic alterations of NRIP1 and their prognostic impact on AML (A,B) OncoPrint from cBioPortal showing NRIP1 mutation profiles (amplifications and deletions) in TCGA AML cohorts. (C) Kaplan-Meier analysis of OS in patients with NRIP1 alterations versus wild-type patients. (D) Disease-free survival (DFS) comparison between groups.

Genetic mutations are among the primary etiological factors in AML [34]. In our study, genomic profiling of two independent AML cohorts via cBioPortal revealed that NRIP1 underwent various genomic alterations, including amplifications and deep deletions, with an overall alteration frequency of 1.3% ( Figure 6A,B). Strikingly, patients harboring these NRIP1 alterations had significantly shorter overall survival than those with wild-type NRIP1 ( *P* = 8.316 × 10 ^–6^), whereas no significant difference was observed in disease-free survival ( *P* = 0.0802) ( Figure 6C,D). These results establish NRIP1 genomic alterations as clinically relevant biomarkers in AML, particularly for predicting overall survival outcomes, and suggest their potential utility in risk stratification and therapeutic decision-making.

### NRIP1 expression is correlated with distinct immune infiltration patterns in AML

**Figure 7:**
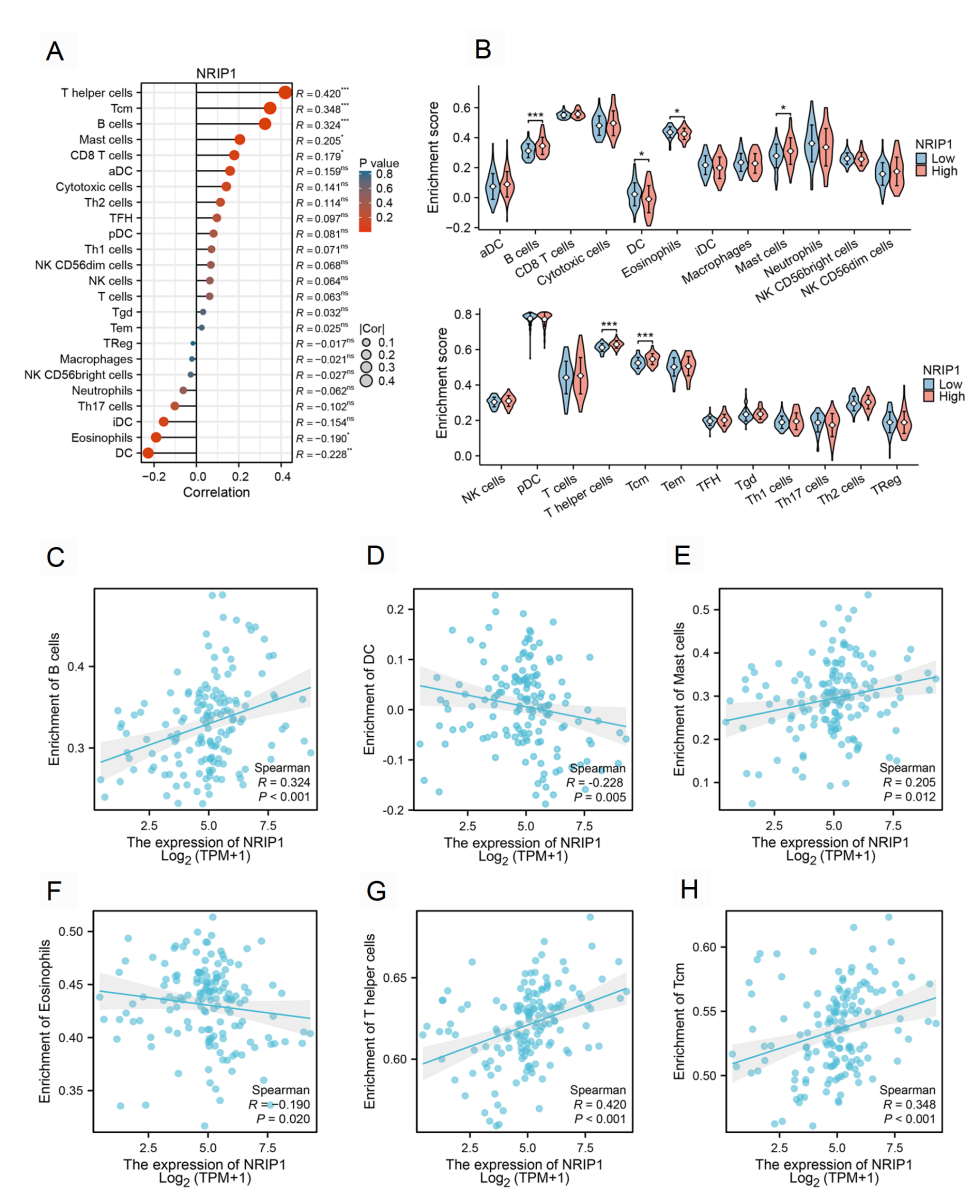
Figure 7 NRIP1 expression is correlated with immune infiltration patterns in AML (A) Bubble plot depicting the Spearman correlation coefficients between NRIP1 mRNA expression levels and the enrichment scores of 24 immune cell subsets in the TCGA-LAML cohort (n = 173). Immune cell enrichment scores were calculated using ssGSEA. The size of each bubble represents the absolute value of the correlation coefficient (R). A positive correlation coefficient means that there is a positive correlation between the two variables, while a negative correlation coefficient means that there is a negative correlation between the two variables. (B) Boxplots comparing immune cell infiltration in the NRIP1-high vs. NRIP1-low groups. (C–H) Scatter plots validating associations between NRIP1 and B cells (C), DCs (D), mast cells (E), eosinophils (F), T helper cells (G) and Tcm (H). *P < 0.05, ***P < 0.001.

Given the critical role of immune networks in leukemia biology [35], we systematically evaluated the relationship between NRIP1 expression and tumor immune microenvironment composition via the ssGSEA and CIBERSORT algorithms. We observed NRIP1 expression was significantly correlated with the abundance of multiple immune cell subsets ( Figure 7). Specifically, high NRIP1 expression correlated significantly with increased infiltration of T helper cells (R = 0.420, *P*  < 0.001), central memory T cells (Tcm, R = 0.348, *P*  < 0.001), B cells (R = 0.324, *P*  < 0.001), and mast cells (R = 0.205, *P* = 0.012). Conversely, elevated NRIP1 levels were negatively associated with eosinophils (R = –0.190, *P* = 0.02) and dendritic cells (DCs, R = –0.228, *P* = 0.005). In summary, NRIP1 expression is associated with a distinct immune cell infiltration profile in the AML microenvironment.

### NRIP1 inhibition suppresses AML cell proliferation and induces apoptosis

**Figure 8:**
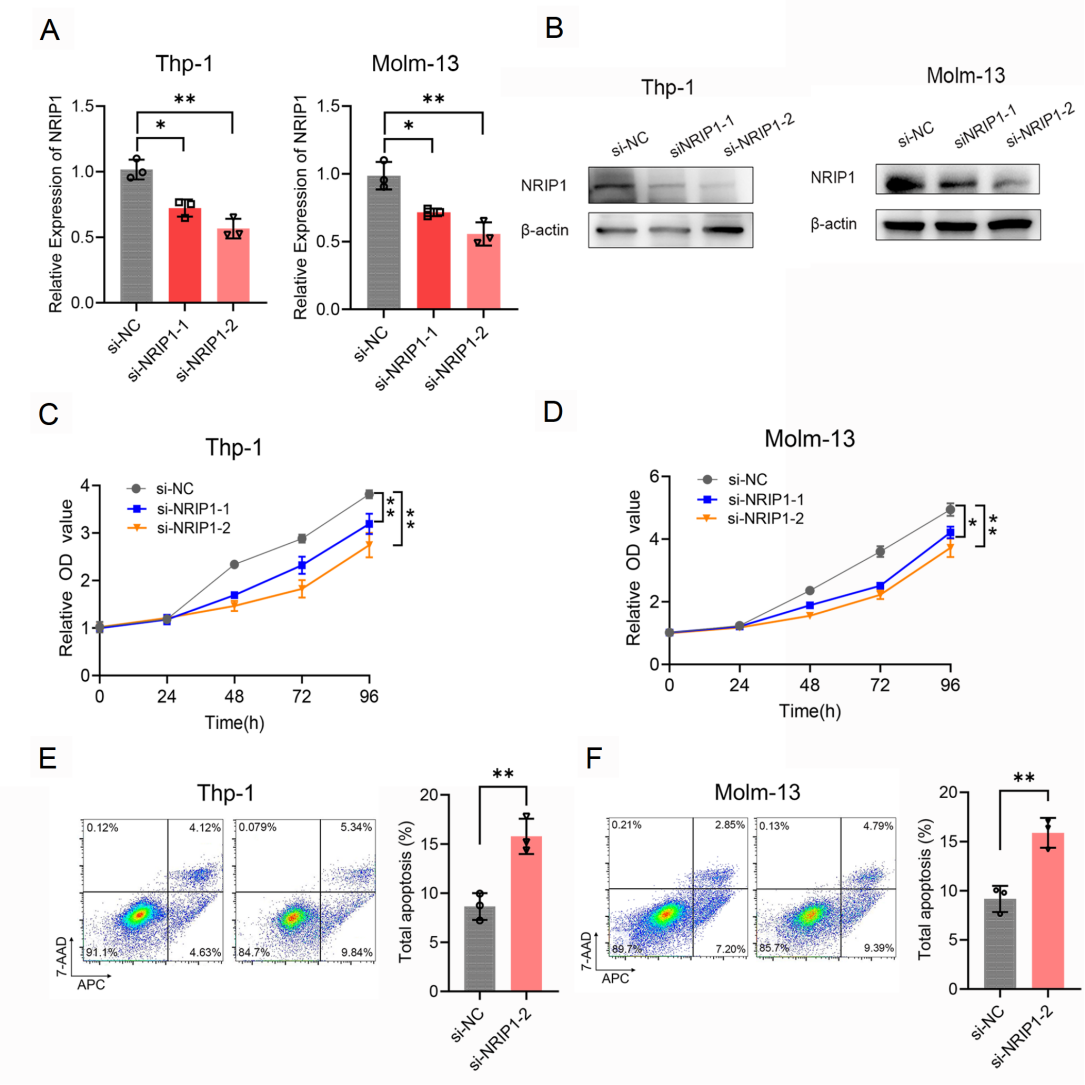
Figure 8 *NRIP1* knockdown suppresses AML cell proliferation and induces apoptosis (A,B) qRT-PCR and western blot analysis confirmed the efficiency of NRIP1 knockdown in Thp-1 and Molm-13 cells transfected with siNRIP1-1, siNRIP1-2, or scrambled control (si-NC). (C,D) CCK-8 assays demonstrating reduced proliferation in NRIP1-depleted cells (96 h). (E,F) Flow cytometry analysis of apoptosis (Annexin V+/PI− and Annexin V+/PI+ populations) 48 h posttransfection. Data are presented as the mean ± SEM (n = 3). *P < 0.05, **P < 0.01.

To investigate the oncogenic role of NRIP1 in AML cell survival, we knocked down *NRIP1* in two representative AML cell lines, Thp-1 and Molm-13. Compared with cells transfected with a nontargeting scrambled control (si-NC), cells transfected with two independent NRIP1-specific siRNAs (siNRIP1-1 and siNRIP1-2) effectively downregulated NRIP1 expression at both the mRNA and protein levels, confirming the efficiency of our genetic intervention ( Figure 8A,B). Then we evaluated the functional consequences of NRIP1 depletion on cell proliferation. Using CCK-8 assays, we monitored cell viability over a 96-h period. *NRIP1* knockdown resulted in a significant and time-dependent suppression of proliferation in both cell lines ( Figure 8C,D). At the 96-h time point, the viability of *NRIP1*-knockdown cells was significantly lower than that of the si-NC-transfected cells, indicating that NRIP1 is essential for sustaining the proliferative capacity of AML cells. We then sought to determine whether the observed anti-proliferative effect is driven by the induction of programmed cell death. Apoptosis was quantified by flow cytometry using Annexin V-FITC/PI dual staining at 48h post-transfection. Consistent with the proliferation data, *NRIP1* silencing markedly induced cell apoptosis. Flow cytometric analysis revealed a significant increase in apoptosis (Annexin V ^+^ cells) following *NRIP1* knockdown compared with that of the controls ( *P* <0.01, Figure 8E,F). In conclusion, NRIP1 has a critical effect in regulating cell apoptosis and maintaining cell proliferation in AML.

## Discussion

Our study identified NRIP1 as a multifunctional regulator and prognostic biomarker in AML. We demonstrated that NRIP1 is significantly overexpressed in AML patients and cell lines and that its elevated expression is strongly associated with adverse clinical features and poor overall survival. Through integrated bioinformatics and functional validation, we established its role in modulating key oncogenic processes, including the suppression of apoptosis, the regulation of ferroptosis, and the impact on the TME. These results highlight the importance of NRIP1 in AML pathogenesis and its potential as a therapeutic target.

Originally characterized as a nuclear receptor coregulator [36], NRIP1 has since emerged as a multifaceted oncogenic driver across malignancies. While its tumor-promoting role has been well documented in solid tumors, including breast [37], colorectal [38], and, more recently, thyroid [39] cancers, its biological significance in AML has remained elusive. Our pan-cancer analysis of TCGA data not only confirmed NRIP1 overexpression in multiple cancer types (breast, esophageal, and thyroid carcinomas) but also established its clinical relevance in AML. Notably, in addition to confirming its overexpression, we provide evidence that links NRIP1 to immune infiltration patterns and ferroptosis regulation in AML. The molecular landscape of AML is fundamentally shaped by genetic alterations [40], with recent evidence implicating dysregulated ferroptosis as both a pathogenic mechanism and a therapeutic opportunity [ 41, 42] . While immune-based therapies have revolutionized cancer treatment [43], their application in AML remains limited. This is largely due to the profoundly immunosuppressive bone marrow microenvironment characteristic of the disease [ 44, 45] . Our data indicate that NRIP1 expression is associated with an immunosuppressive microenvironment, characterized by specific patterns of immune cell infiltration. Furthermore, the coordinated regulation of ferroptosis drivers (ALOX15) and suppressors (GPX4 and SLC7A11) by NRIP1, validated by protein-level changes upon *NRIP1* knockdown, reveals a previously unappreciated role for NRIP1 in modulating ferroptosis.

Functionally, our data suggest that NRIP1 is a regulator that co-ordinates multiple oncogenic processes, with notable enrichment in glycoprotein metabolic processes. These findings suggest that NRIP1-driven remodeling of the glycoproteomic landscape may directly impact the glycosylation, functionality, and drug sensitivity of key oncogenic drivers such as FLT3 [46]. Therefore, combining NRIP1-targeting strategies with FLT3 inhibitors or glycosylation-modulating drugs represents a promising synergistic therapeutic approach. PPI network analysis revealed functionally significant interactions between NRIP1 and key regulatory proteins, notably NR3C1 (glucocorticoid receptor) and RORA (nuclear receptor-related orphan receptor alpha). NR3C1, which has been shown to promote ovarian tumor metastasis [47] and is implicated in chemotherapy resistance [48], has also been reported to inhibit erastin-induced ferroptosis in breast cancer [49] and predict the prognosis and tumor microenvironment in endometrial cancer [50]. RORA primarily influences immune evasion in melanoma by modulating PD-L1 transcription [51]. Collectively, these functional interactions not only illustrate how NRIP1 influences tumor development and immune evasion but also raise important clinical considerations.

An important clinical question is whether NRIP1 is itself a druggable target. Currently, no direct NRIP1 inhibitors are clinically available. This presents a significant challenge but also an opportunity for drug discovery. However, given its role as a coregulator for nuclear receptors such as RARA and ESR1, proteins targeted by established therapeutics ( *e*. *g*., ATRA and tamoxifen), an indirect targeting strategy appears feasible. Repurposing these drugs to inhibit the NRIP1 complex warrants investigation. Among the 25 ferroptosis-related genes analyzed, NRIP1 expression was significantly positively correlated with 12 and negatively correlated with the other 12 genes, while no significant association was detected with the remaining genes. These findings suggest that NRIP1 may be involved in the ferroptosis process. Given that venetoclax resistance mechanistically involves impaired ferroptosis [52], targeting this axis represents a potential strategy to overcome treatment resistance.

Despite the compelling associations revealed by our integrated analysis, this study has several limitations that warrant acknowledgement. First, while our *in vitro* experiments demonstrated a functional role for NRIP1 in proliferation and apoptosis, the proposed mechanisms regulating the immune microenvironment remain based primarily on computational inference. Future work employing co-culture systems of AML cells with primary immune cells is essential to experimentally validate the immunomodulatory role of NRIP1. Second, the link between NRIP1 and ferroptosis requires direct validation through measurements of lipid ROS, glutathione (GSH) levels, and mitochondrial health upon NRIP1 modulation. Finally, further prospective validation in a larger, multi-center cohort or protein array will be invaluable to establish the clinical utility of NRIP1 for risk stratification in AML patients.

In conclusion, our study provides a comprehensive analysis of the relationship between NRIP1 and AML. We demonstrated its significant overexpression and association with poor prognosis and implicated it in immune infiltration. These findings suggest that NRIP1 is a promising diagnostic and prognostic biomarker and a potential target for immunotherapy in AML.

## Supporting information

25719Supplementary_Table_S1

25719Supplementary_Table_S2

25719Supplementary_Figures

## Supplementary Data

Supplementary data is available at *Acta Biochimica et Biophysica Sinica* online.
