# Supplementary material for: The prognostic marker NRIP1 is associated with tumor progression and immune infiltration in acute myeloid leukemia: NRIP1 is associated with tumor progression and immune infiltration
Source: Acta Biochim Biophys Sin (Shanghai). 2025 Nov 4;58(2):437–52. doi: 10.3724/abbs.2025197 (PMC12900777; doi:10.3724/abbs.2025197)
Supplement: 25719Supplementary_Figures [file 25719Supplementary_Figures.docx]

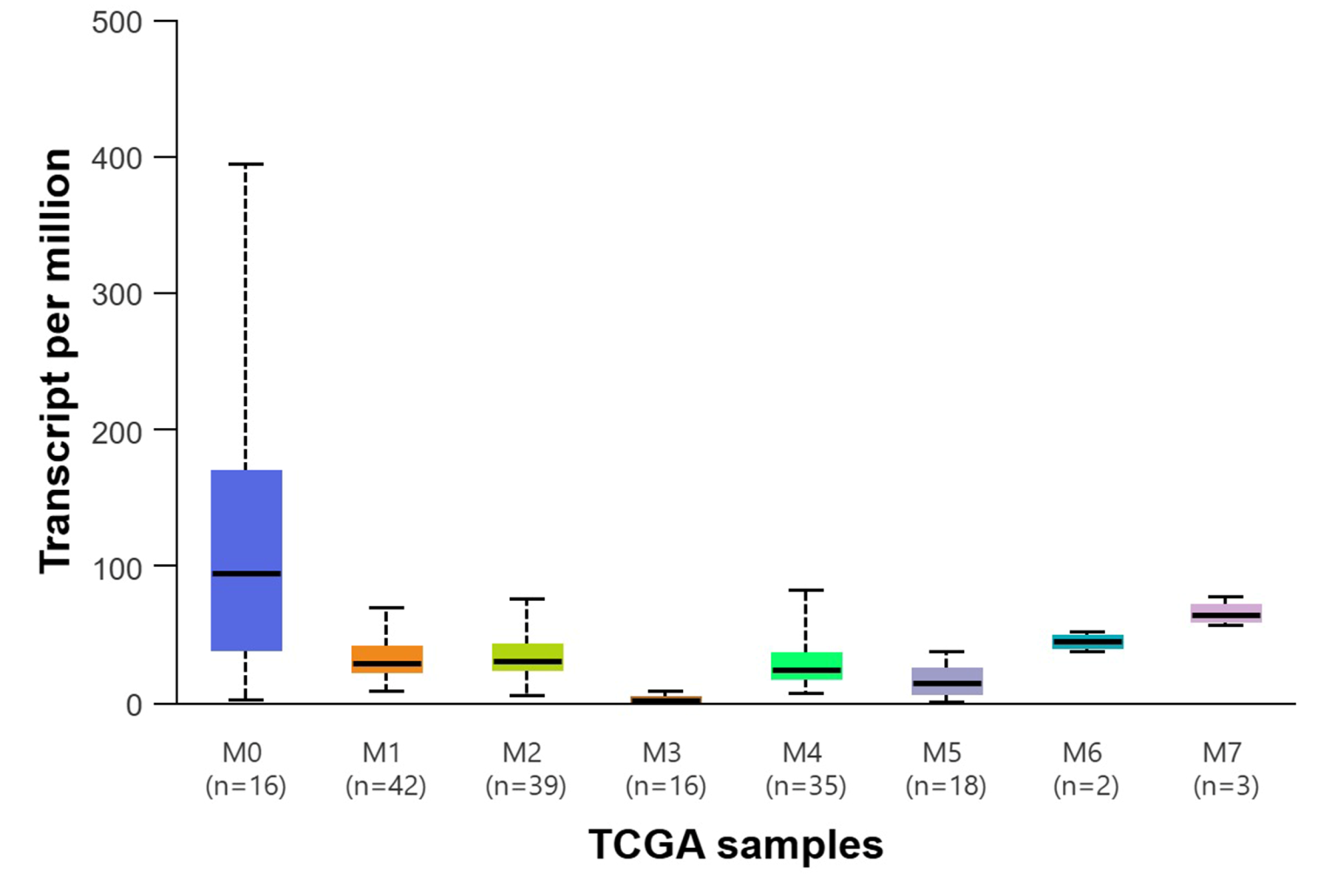


**Supplementary Figure S1. Expression of NRIP1 in LAML based on French American British classification**

**
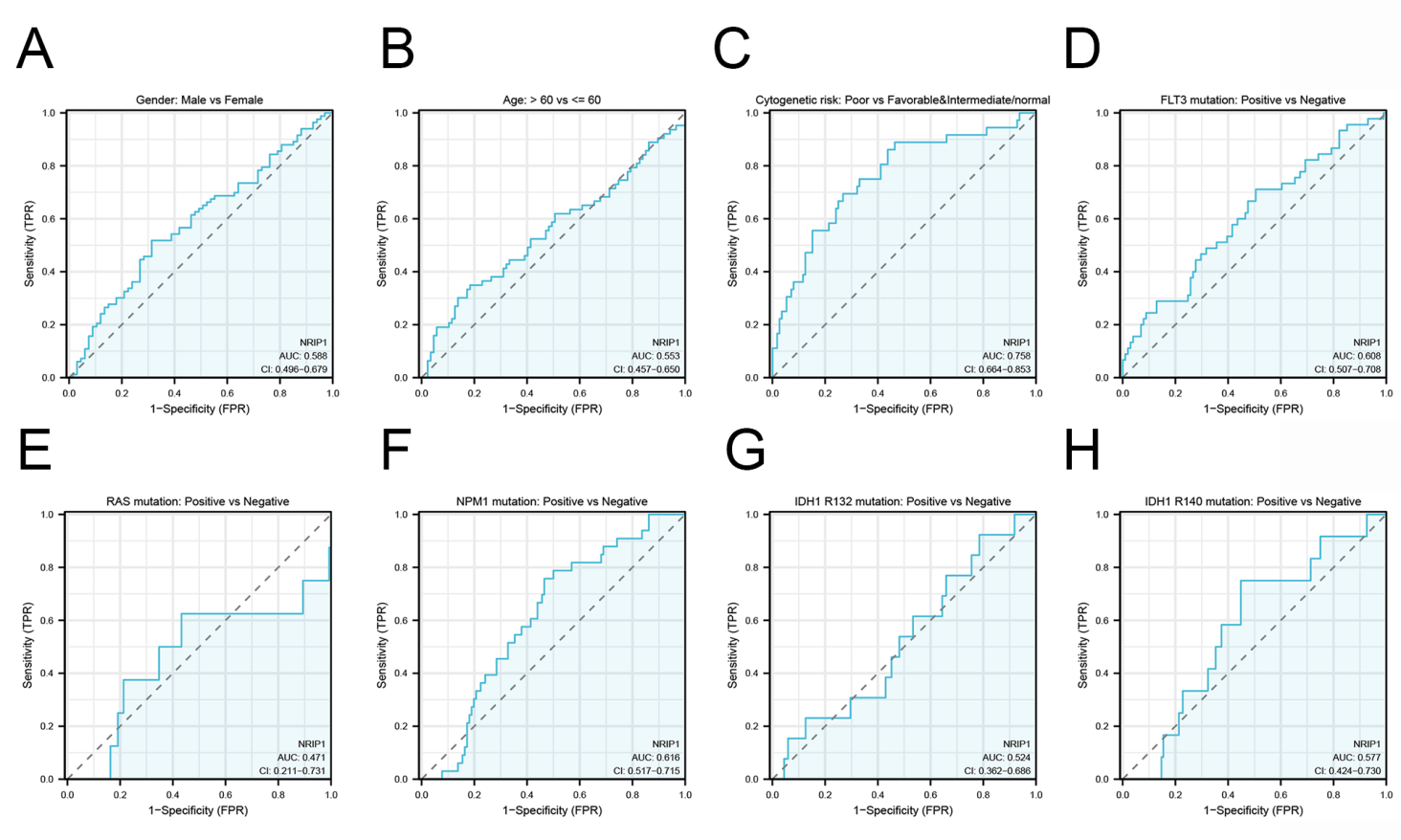
**

**Supplementary Figure S2. ROC validation of NRIP1 in different clinicopathological features** Diagnostic ROC curves in (A) Gender, (B) Age, (C) Cytogenetic risk, (D) FLT3 mutation, (E) RAS mutation, (F) NPM1 mutation, (G) IDH1 R132 mutation, and (H) IDH1 R140 mutation.


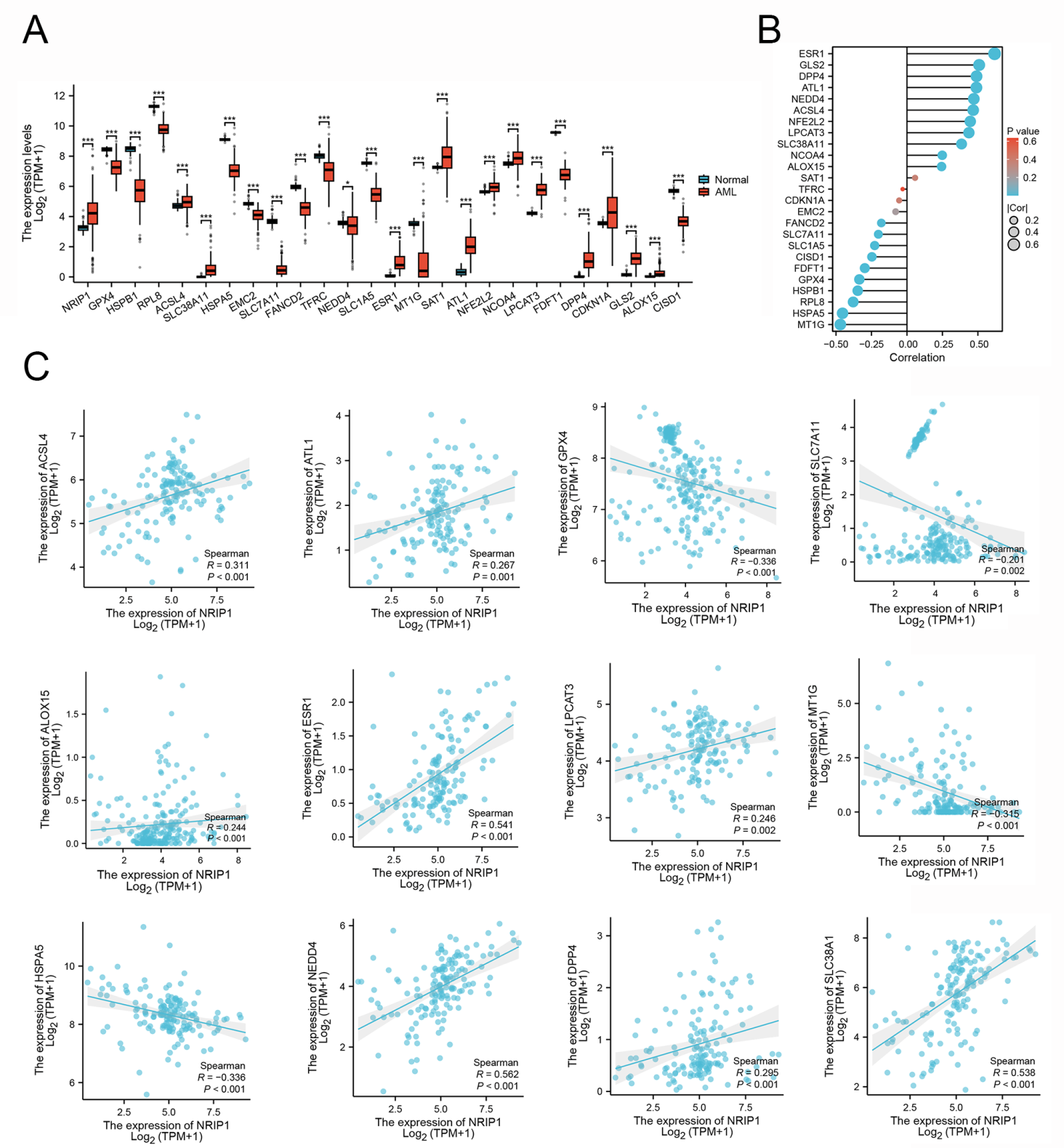


**Supplementary Figure S3. Correlation of NRIP1 expression with ferroptosis-related genes** (A) Analysis of ferroptosis-related genes between high and low groups of NRIP1 in TCGA and GTEx data. (B) Bubble plot of the correlation between NRIP1 and 25 ferroptosis-related genes. (C) Scatter plots of correlation between NRIP1 expression levels and ferroptosis-related genes (｜R｜≥ 0.2 ). ns: *P* ≥ 0.05, **P* < 0.05, ***P* < 0.01, and ****P* < 0.001.
